# Artificial intelligence across oncologic theranostics: evidence for patient stratification, dosimetry, and adaptive radiopharmaceutical therapy

**DOI:** 10.3389/fnume.2026.1935247

**Published:** 2026-08-27

**Authors:** Mallareddy Banala, Shabbir Ezuddin, Mark Foley, Russ Kuker

**Affiliations:** Department of Radiology, University of Miami Miller School of Medicine and Jackson Health System, Miami, FL, United States

**Keywords:** adaptive treatment, artificial intelligence, dosimetry, PRRT, PSMA, radioligand therapy, radiopharmaceutical therapy, theranostics

## Abstract

Artificial intelligence (AI) has been studied across radiopharmaceutical therapy (RPT); however, evidence for treatment-changing use remains limited. We conducted a structured narrative review with descriptive mapping of learned models for patient stratification, segmentation, tumor-burden quantification, quantitative preprocessing, dosimetry, toxicity prediction, response assessment, and radiation-safety/logistics support. The mapped set contained 73 direct full journal reports and eight direct conference abstracts, with one additional meeting abstract retained as contextual evidence. Among the full reports, 17 were PSMA-related, 16 SSTR/PRRT, 15 radioiodine, 14 ⁹⁰Y radioembolization, and 11 cross-platform or emerging-target reports; 16 addressed segmentation or quantification, 32 selection, response, prognosis, toxicity, or safety/logistics, and 25 registration, preprocessing, or dosimetry. Fifty-one reports were retrospective, one was an explicitly prospective clinical/technical evaluation, nine were technical, synthetic, or phantom evaluations, and temporal design was unclear or conflicting in 12. Seven reported an external-type held-out evaluation, including four that clearly held out an institution; one reported explicit calibration, one propagated task-level uncertainty, and none evaluated a prospective AI-guided treatment policy. The mapped evidence most directly supports human-reviewed measurement and workflow assistance. An eight-domain RPT evidence-to-decision synthesis organizes quantitative fidelity, reference standards, validation, endpoints, biological linkage, oversight, uncertainty and quality assurance, and decision impact; it is not a validated score or adoption standard.

## 1 Introduction: from fixed-activity workflows to AI-supported precision theranostics

Radiopharmaceutical therapy (RPT) has rapidly expanded in clinical settings. Prostate-specific membrane antigen (PSMA)-targeted radioligand therapy (RLT) for prostate cancer, somatostatin receptor (SSTR)-targeted peptide receptor radionuclide therapy (PRRT) for neuroendocrine tumors, radioiodine therapy for thyroid cancer, and ⁹⁰Y radioembolization illustrate the theranostic principle of imaging the target, treating the target, quantifying the biodistribution, absorbed dose, and subsequent response (1–7). Despite this conceptual precision, many workflows still use fixed or empirically administered activities and require labor-intensive segmentation, serial imaging, activity quantification, time-activity modeling, and absorbed-dose reporting.

AI is attractive in this setting because the RPT workflow is data-rich and quantitatively constrained. Patients may undergo baseline positron emission tomography/computed tomography (PET/CT) or single-photon emission computed tomography/computed tomography (SPECT/CT), post-therapy quantitative imaging, serial laboratory testing, symptom and toxicity monitoring, and repeated treatment cycles. These data can be linked to activity concentration, time-integrated activity (TIA), absorbed dose, effective half-life, tumor burden, response, and clinical measures of organ reserve. AI methods may reduce the segmentation burden, standardize tumor-burden quantification, infer kinetic parameters, approximate computationally expensive dose calculations, and estimate response or toxicity risk (8–15).

The central clinical question is not whether AI can produce a high-performing model in a retrospective dataset, but whether an AI-supported output can safely and reproducibly improve treatment decisions. Relevant decisions include whether to treat, whether to intensify or de-escalate activity, whether to delay or discontinue a cycle, whether the predicted organ dose is acceptable, and whether early response suggests continued benefit. Discrimination alone is insufficient when a model is small, single-center, poorly calibrated, vulnerable to data leakage or dataset shift, or untested for decision impact (11, 14, 16–19).

We organized the evidence around the clinical theranostic continuum rather than algorithm categories. We examined AI for treatment stratification and response assessment, automated segmentation and tumor burden quantification, personalized dosimetry, toxicity prediction, and response-adaptive planning. We distinguished technical feasibility from clinical utility, segmentation accuracy from dosimetric accuracy, dosimetric agreement from outcome improvement, and conceptual digital twin frameworks from clinically tested systems (14–16, 20–22).

## 2 Evidence identification and review approach

Using access provided by the University of Miami Libraries, including the Louis Calder Memorial Library, we searched PubMed/MEDLINE, Embase (Embase.com), Scopus, and Web of Science Core Collection. Searches were run on July 18, 2026. PubMed used an exact publication-date interval from January 1, 2015, through July 18, 2026; Embase, Scopus, and Web of Science used the available 2015–2026 publication-year limits, so those results comprise records indexed and retrievable when the searches were run. No language, human-only, publication-type, or full-text filter was applied. The searches returned 1,047, 1,106, 5,591, and 1,017 records, respectively (8,761 before deduplication). Conflict-aware sequential matching by canonical DOI, PMID, and exact normalized title removed 24 within-source and 2,380 additional cross-database duplicates, leaving 6,357 unique records. These figures describe electronic identification and deduplication; they do not imply manual screening of all 6,357 records. Exact strategies and safeguards for ambiguous identifiers are reported in Supplementary File 1.

This was a structured narrative review with descriptive evidence mapping, not a systematic review, scoping review, or meta-analysis; no protocol was registered. It was designed to characterize clinically relevant applications, evidentiary limitations, and validation priorities rather than estimate pooled effects or claim exhaustive identification of every eligible report. The 2015 start date captured the contemporary deep learning and modern clinical RPT period; earlier trials, standards, and dosimetry guidance were retained purposively as context. The Scale for the Assessment of Narrative Review Articles (SANRA) domains structured reporting of the review question, search, referencing, reasoning, and presentation and were not used as a study-level risk-of-bias instrument (23).

A report was classified as direct AI evidence when it evaluated a model learned from data: neural networks, random forests or other tree ensembles, gradient-boosting methods, support-vector or other kernel methods, naïve Bayes, nearest-neighbor, or comparable machine-learning classifiers, or trained automated segmentation. A radiomics report entered the direct-AI map only when its primary predictive model belonged to one of these learned method families. Hybrid pipelines were counted as direct learned-model evidence only when the qualifying learned model itself was trained and quantitatively evaluated for an eligible RPT outcome; any downstream conventional regression or nomogram was labeled separately. A learned method used only for variable selection, without separate learned-model performance, did not qualify. Conventional linear, logistic, or Cox regression, penalized regression used within a conventional statistical pipeline, discriminant analysis, nomograms, and radiomics association studies without such a learned model were classified as statistical or quantitative context. Hand-engineered quantitative imaging without a predictive model, reconstruction, dosimetry, computational fluid dynamics, guidelines, and software papers were also contextual. Records were eligible when they addressed an oncologic RPT decision or a measurement feeding that decision: treatment stratification, response assessment, lesion or organ segmentation, tumor-burden quantification, patient-specific dosimetry, toxicity prediction, radiation-safety or treatment-logistics prediction, or adaptive planning. Generic diagnostic imaging AI and external-beam radiotherapy AI were excluded unless directly informative for an RPT task. One exploratory diagnostic FAPI dose-rate-map report was retained in the emerging quantitative-measurement category because it learned a voxel map proposed as a technical input to future therapeutic dosimetry; its inclusion does not constitute validation for a therapeutic FAPI radionuclide or treatment decision.

Reports were selected purposively when they met the direct learned-model definition; backward and forward citation searching identified related reports and contextual standards. Because a complete prospective log of title/abstract exclusions, full-text assessments, exclusion reasons, and citation-chasing yields was not maintained, these quantities were not reconstructed retrospectively. Two authors used a sequential verification process for the retained narrative evidence set. M.B. reviewed candidate titles and abstracts, assessed candidate reports for eligibility, completed structured extraction, and verified numerical claims. S.E. subsequently verified retained-report eligibility, publication type, platform, and task classification, and extracted numerical claims against the available source records. This workflow did not constitute independent duplicate screening or extraction of all 6,357 unique electronic records. Questions were resolved through discussion and source rechecking by M.B. and S.E.; no third-author adjudication or formal agreement statistic was used. M.F. and R.K. reviewed the clinical interpretation and revised the manuscript. Conference abstracts were eligible only when publication status and an official source were verifiable and no superseding full report was identified; they were labeled explicitly and analyzed separately from full reports. The eight direct abstracts were excluded from the 73-report denominator; the Djaileb meeting abstract was retained only as contextual evidence. Report family and possible cohort overlap were recorded rather than treated as independent cohorts.

For retained reports, the structured inventory recorded the publication type, cohort and unit of analysis, tracer and input data, intended use, reference standard, comparator, internal or external validation, calibration or uncertainty reporting, principal metric, and connection between model output and clinical action when these attributes were available in the audited source. The full text was available for 67 of the 73 mapped direct journal reports; extraction for six journal records was constrained to an official abstract or publisher preview and is flagged in Supplementary Section S7.1. The term full journal report denotes a publication type rather than a uniform source access. Fields that were not systematically extracted were labeled as such rather than being converted to a claim that the primary report omitted them. Multicenter data were considered for external validation only when a center, scanner environment, or appropriate temporal cohort was held out from the model development. Main-text examples were selected for external or prospective testing, a clinically relevant endpoint, explicit quantitative comparison with reference dosimetry, a comparatively informative cohort within a platform–task group, or illustration of recurrent methodological limitations. Supplementary Sections S7.1–S8 provide the study-level inventory, metrics, evidence role labels, and descriptive characterizations. Supplementary File 1 lists all 73 direct reports and 72 retained contextual/non-direct sources: 70 numbered manuscript references outside the direct full-report set and two supplementary-only contextual records.

Descriptive methodological characterization, rather than a formal risk-of-bias appraisal or pooled score, was informed by PROBAST + AI concepts for prediction-model studies, Radiomics Quality Score 2.0 (RQS 2.0) domains for radiomics studies, and QUADAS-3 domains for diagnostic-accuracy designs, where applicable. Segmentation and dosimetry studies were characterized by reference-standard construction, patient-level splitting, external testing, quantitative consequences, uncertainty, and decision linkage (24–26). Inapplicable domains were marked accordingly. When reporting was insufficient for an examined domain, the judgment was recorded as unclear; fields that were not systematically extracted remain explicitly labeled “not separately extracted.” A descriptive characterization table is provided in Supplementary File 1.

To examine the clarity and task applicability of the eight-domain RPT evidence-to-decision synthesis, we applied it descriptively to a purposively selected six-report pilot comprising two prediction/classification, two segmentation/localization, and two dosimetry or dose-map-prediction reports. Each study domain pair was assigned a source-anchored status of Yes, Partial, No, or Not applicable for the narrowly evaluated intended use. The 48 non-scoring judgments comprised 2 Yes, 28 Partial, 16 No, and 2 Not applicable responses. The pilot was not a formal risk-of-bias assessment, representative sample, validated instrument, or basis for a composite score or an adoption threshold. Supplementary Section S10.2 provides definitions, study-level rationales, and physical source-page anchors.

The direct learned-model map contained 73 full journal reports and eight direct conference abstracts analyzed separately; one additional meeting abstract was retained as contextual evidence. Among the full reports, 17 were PSMA-related, 16 SSTR/PRRT-related, 15 radioiodine-related, 14 ⁹⁰Y radioembolization-related, and 11 cross-platform or emerging-target-related. Sixteen addressed segmentation or quantitative burden, 32 selection, response, prognosis, toxicity, or safety/logistics, and 25 addressed registration, preprocessing, or dosimetry. Fifty-one reports were explicitly retrospective, one was an explicitly prospective clinical/technical evaluation, nine were technical, synthetic, or phantom evaluations, and the temporal design was unclear or conflicting in 12. The center status was single-source or one center in 41 reports (including one single-system phantom report), multi-institutional or bicentric in 10, and unclear or not applicable in 22. Seven reported an external-type held-out evaluation, and four clearly held out an institution. One reported explicit calibration, one propagated task-level uncertainty, and none evaluated calibrated predictive uncertainty with coverage or out-of-distribution performance. No report prospectively evaluated an AI-guided treatment policy or patient-outcome decision impact. Counts describe reports in the mapped narrative set, not unique cohorts or prevalence in an exhaustive systematic review population.

## 3 The AI-supported theranostic data ecosystem

**Figure 1:**
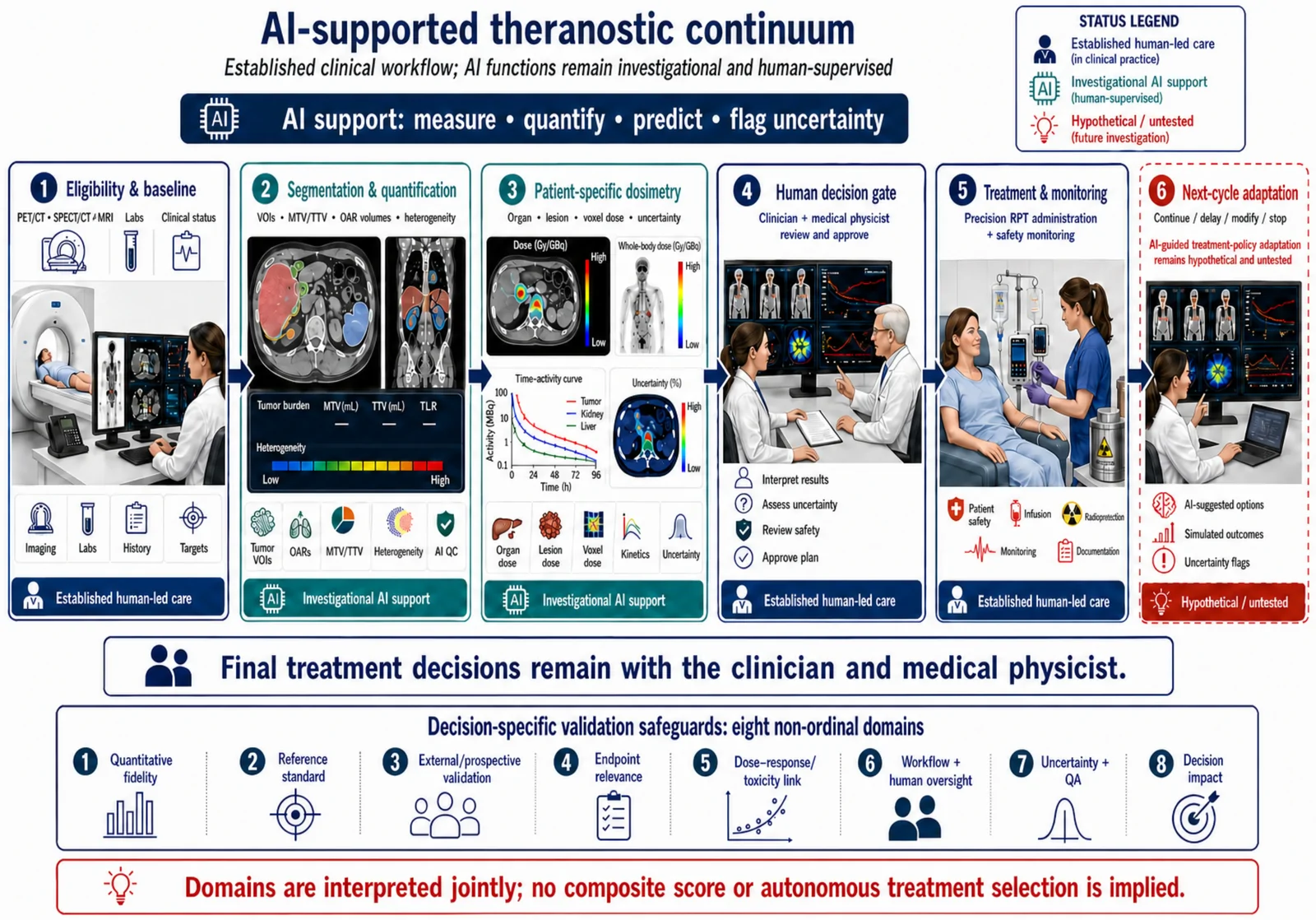
AI-supported theranostic continuum and validation safeguards. Baseline molecular and cross-sectional imaging, laboratory data, clinical history, target expression, disease burden, and organ reserve inform eligibility and treatment planning. Human-reviewed lesion and organ-at-risk segmentation supports quantitative characterization and, when performed, patient-specific dosimetry. Clinician and medical-physicist review integrates measured and model-derived quantities before treatment delivery, longitudinal monitoring, and protocol-defined next-cycle decisions. AI supports measurement, quantification, prediction, and quality assurance; it does not make the final treatment decision. The eight interpretive domains act as cross-cutting safeguards: quantitative fidelity; appropriateness of the reference standard; external or prospective validation; clinical-endpoint relevance; dose-response or dose-toxicity linkage; workflow fit and human oversight; uncertainty and quality assurance; and decision impact. AI, artificial intelligence; CT, computed tomography; MRI, magnetic resonance imaging; PET, positron emission tomography; QA, quality assurance; RPT, radiopharmaceutical therapy; SPECT, single-photon emission computed tomography; TIA, time-integrated activity. The status coding distinguishes established human-led care, investigational AI support, and hypothetical or untested treatment-changing adaptation. Created in BioRender. Banala, M. (2026) https://BioRender.com/hjo0hkn.

**Figure 2:**
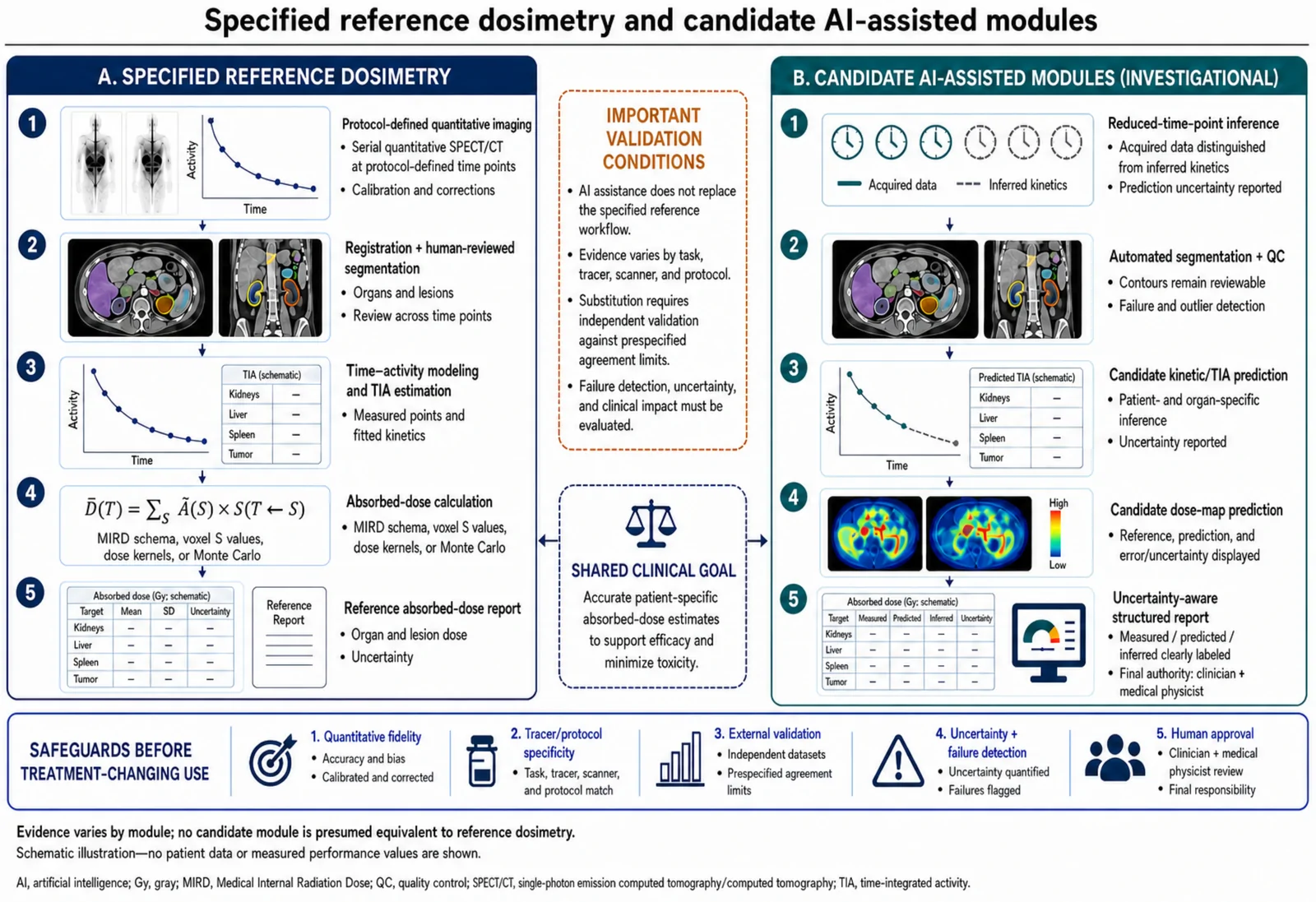
Specified reference dosimetry and candidate AI-assisted modules. A specified reference workflow comprises protocol-defined quantitative imaging, calibration and corrections, registration, human-reviewed segmentation, time-activity modeling and time-integrated activity estimation, and absorbed-dose computation using an explicit dosimetry formalism, such as the MIRD schema, voxel S values, dose-kernel convolution, or Monte Carlo simulation. Candidate AI modules may assist segmentation, reduced-time-point kinetic inference, dose-map prediction, computational surrogates, and structured reporting; the supporting evidence differs by task, and no module is assumed equivalent to the reference workflow. Measured, predicted, and inferred quantities should be distinguished, uncertainty should be reported, and substitution requires independent validation showing agreement within prespecified clinically acceptable limits for the intended tracer, scanner, protocol, organ or lesion, and decision. All panels are schematic illustrations; imaging time points are protocol dependent, and no patient data or measured performance values are shown. Final interpretation remains with the clinician and medical physicist. AI, artificial intelligence; Gy, gray; MIRD, Medical Internal Radiation Dose; QC, quality control; SPECT/CT, single-photon emission computed tomography/computed tomography; TIA, time-integrated activity. Created in BioRender. Banala, M. (2026) https://BioRender.com/5b04skc.

RPT is a closed-loop quantitative pathway: baseline imaging and clinical data characterize target expression, disease burden, and organ reserve; post-therapy imaging estimates biodistribution and absorbed dose; and interval response, laboratory trends, and toxicity inform subsequent cycles (Figure 1). Candidate AI roles include human-reviewed segmentation and tumor-burden measurement, quantitative preprocessing, reduced-timepoint or voxel dosimetry, response and toxicity prediction, and longitudinal decision support (Figure 2). Their evidentiary requirements differ: geometric accuracy does not establish quantitative fidelity, prognostic discrimination does not identify treatment benefit, and dose estimation does not justify activity modification without an actionable biological relationship and tested management rule. Emerging targets should remain visible but proportionate. FAP-targeted therapy has entered early nonrandomized therapeutic evaluation, including a first-in-human [^1^⁷⁷Lu]Lu-EB-FAPI study and the multicenter phase I LuMIERE study of [^1^⁷⁷Lu]Lu-FAP-2286; neither evaluated an AI-guided policy (27, 28). Alpha-, Auger-, CXCR4-, and other emerging platforms likewise provide research context, whereas direct treatment-linked AI evidence remains sparse (1–11, 20–22, 29–48).

## 4 RPT evidence-to-decision synthesis

We synthesized eight non-ordinal RPT evidence-to-decision domains: quantitative fidelity, reference-standard appropriateness, external or prospective validation, clinical-endpoint relevance, dose-response or dose-toxicity linkage, workflow and human oversight, uncertainty and quality assurance, and decision impact. Evidence stage is reported separately from these domains (17–19).

This synthesis is a narrative organizing aid, not a new validated framework, local-adoption tool, deployment threshold, certification scheme, or score; no inter-rater reliability or clinical validity is claimed for it.

The domains were derived by mapping recurrent gaps across the retained evidence and comparing them with RELAINCE, RQS 2.0, CLAIM, TRIPOD + AI, PROBAST + AI, and DECIDE-AI. The RPT-specific emphasis is the separation of dosimetric fidelity, biological linkage, and treatment action; the synthesis does not replace established reporting or evaluation guidance. Supplementary File 1 provides the side-by-side mapping, task-specific descriptive characterization approach, and a six-report non-scoring pilot application (17–19, 24, 25, 49).

Illustrative evaluation considerations scale with intended-use risk: human-reviewed contour pre-population requires local fidelity and failure testing; dosimetry triage additionally requires external validation, measurable uncertainty, and prospective workflow evaluation; treatment selection or modification requires validated treatment-effect or dose-response logic and prospective multicenter testing of a pre-specified rule. These are research design considerations, not adoption criteria.

**Figure 3:**
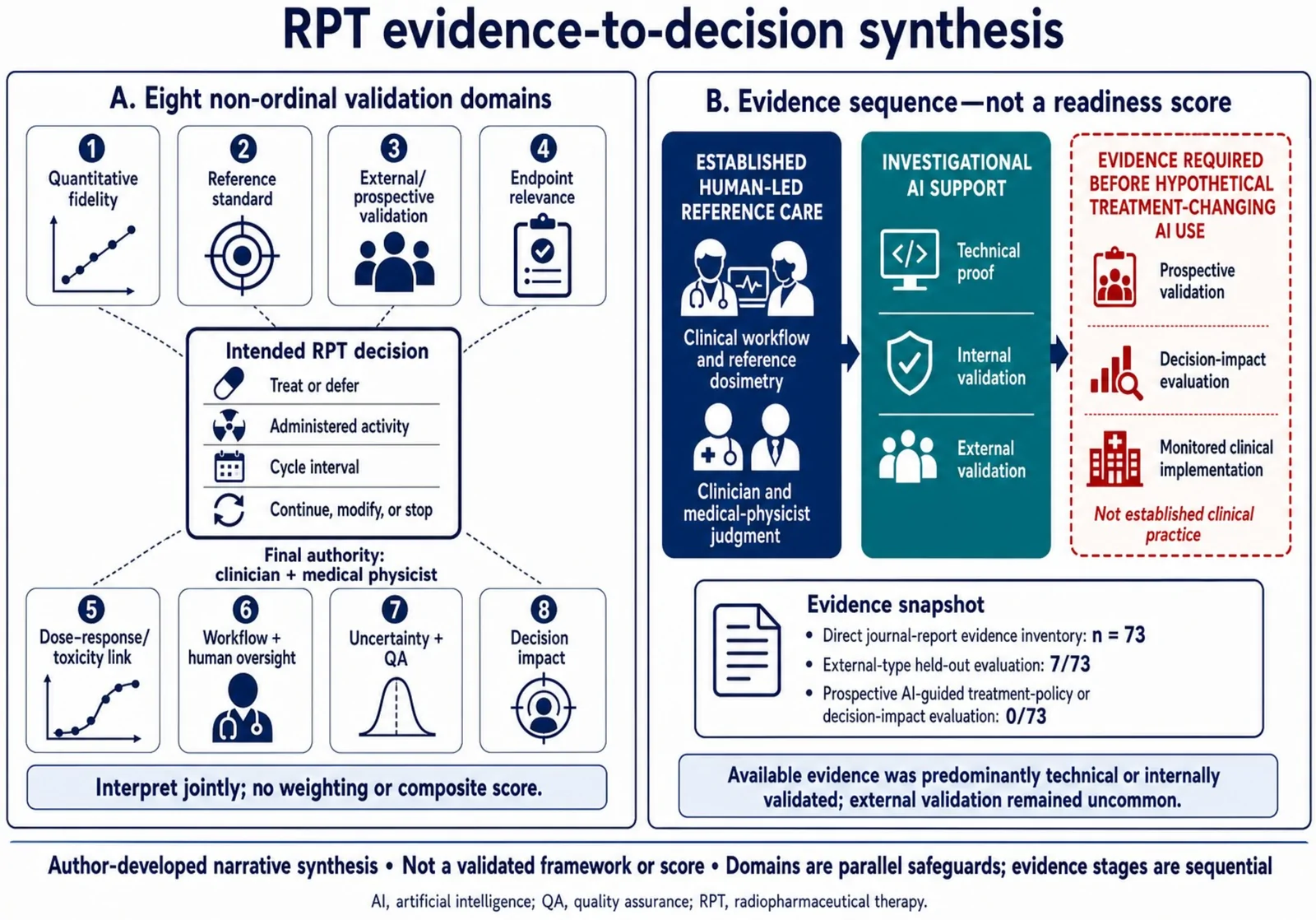
RPT evidence-to-decision synthesis. Panel **(A)** presents eight non-ordinal domains considered in parallel: quantitative fidelity, appropriateness of the reference standard, external or prospective validation, clinical-endpoint relevance, dose-response or dose-toxicity linkage, workflow fit and human oversight, uncertainty and quality assurance, and decision impact. The domains are interpretive criteria and are not intended to be summed into a composite score. Panel **(B)** presents the separate sequential evidence-stage pathway: technical proof of concept, internal validation, external validation, prospective validation, decision-impact evaluation, and monitored clinical implementation. The domains can be revisited at each stage. Most applications in the mapped narrative set have not progressed to prospective validation or decision-impact evaluation. This is an author-developed narrative synthesis, not a validated framework or scoring instrument. AI, artificial intelligence; QA, quality assurance; RPT, radiopharmaceutical therapy. The evidence snapshot uses the 73 direct full journal reports as the denominator: seven (7/73) reported external-type held-out evaluation, and none (0/73) prospectively evaluated an AI-guided treatment policy or patient-outcome decision impact; direct conference abstracts and contextual sources are excluded. Created in BioRender. Banala, M. (2026) https://BioRender.com/59oxv0n.

Each domain has observable referents. Quantitative fidelity concerns the preservation of activity, volume, kinetics, and absorbed dose against a specified reference. Validation distinguishes internal development, genuinely held-out external testing, prospective observation, and prospective decision impact. Endpoint relevance distinguishes technical surrogates from responses, toxicity, survival, quality of life, and resource outcomes. Pooled multicenter development is not an external validation unless a center, scanner environment, or appropriate temporal cohort is withheld. Table 1 summarizes the platform-level evidence position without ordinal grades, and Figure 3 separates the cross-cutting domains from the evidence stage sequence (8, 11, 17–19, 32–36, 39–42, 50–55).

**Table 1:** Platform-level evidence position and prioritized validation studies. The characterization is descriptive and non-ordinal; study-level designs, metrics, and source-based characterization are reported in Supplementary Sections S7.1–S8.

Platform	Strongest direct evidence	Principal validation or deployment gap	Prioritized next study
PSMA	Retrospective learned prediction, lesion segmentation, automated tumor-burden quantification, and early dose-prediction or dose-map studies; selected held-out-center technical testing.	No prospectively tested treatment-effect model, independent external reference-dosimetry validation, calibrated toxicity rule, or AI-guided activity-prescription trial.	Multicenter locked-model validation with quantitative and dosimetric reference standards, followed by a safety-constrained decision-impact study.
SSTR/PRRT	Retrospective radiomics-plus-ML, random-survival-forest prognostic, and multimodal deep-learning reports; supervised segmentation workflow evidence; and internally evaluated renal, tumor, and voxel-dose prediction.	Predominantly internal validation; in one workflow study, staff-corrected AI contours formed the reference; prognosis has not established treatment benefit or adaptive-policy value.	Independent-reference multicenter dosimetry equivalence and prospective dose-response or dose-toxicity evaluation.
Radioiodine	Retrospective response, iodine-avidity, expert-decision-imitation, localization, registration, dosimetry, and discharge-logistics models; one report included two external cohorts.	Limited calibration, patient-level independence, external transportability, prespecified agreement margins, and prospective policy evaluation.	Patient-separated multicenter validation followed by a prespecified selection, dosimetry, or logistics decision-impact study.
⁹⁰Y/TARE	Retrospective radiomics-plus-ML response studies and technical attenuation, scatter-correction, and dose-prediction reports.	Small heterogeneous cohorts, limited calibration and external validation, and no prospective management-policy or prescription-equivalence evaluation.	Harmonized multicenter quantitative-imaging study with locked response or toxicity models and a prespecified dosimetric margin.
Emerging and cross-platform RPT	Sparse platform-specific learned evidence, including exploratory diagnostic-FAPI dose-map prediction; mechanistic and digital-twin work remains mainly contextual.	Insufficient therapeutic reference standards, external testing, uncertainty characterization, locked policies, and patient-outcome evidence.	Platform-specific prospective technical validation before controlled testing of any treatment-selection or adaptation policy.

TABLE 1 summarizes evidence at the platform level and contains no individual-study rows; per-study publication type and validation class (internal, temporal, institution-held-out, fully external, or prospective) are reported in Supplementary sections S7.1 (direct full journal reports) and S7.3 (direct conference abstracts), with contextual/non-direct sources in S9. The prospectively enrolled SelectPSMA/Djaileb source is a conference (meeting) abstract with an unreported model family; it is classified as contextual evidence, excluded from the 73 direct full-report and eight direct-abstract denominators, and is not counted as prospective validation or decision-impact evidence.

AI, artificial intelligence; FAPI, fibroblast activation protein inhibitor; ML, machine learning; PRRT, peptide receptor radionuclide therapy; PSMA, prostate-specific membrane antigen; RPT, radiopharmaceutical therapy; SSTR, somatostatin receptor; TARE, transarterial radioembolization.

## 5 AI for treatment stratification, prognostication, and response assessment

AI-supported stratification must be assessed at its intended decision point. Pretreatment eligibility, additional-imaging triage, post-treatment response classification, and prognosis imply different actions; most reported models remain retrospective, internally validated decision-support signals rather than autonomous eligibility rules (39–42, 52). Task-level evidence across theranostic platforms is summarized in Table 2; selected direct learned-model studies of segmentation, registration, quantitative preprocessing, and dosimetry are detailed in Table 3; and selected data-driven studies of treatment stratification, prognostication, response assessment, and safety/logistics are presented in Table 4.

**Table 2:** Current evidence position of AI applications across theranostic platforms. Evidence characterization is descriptive and non-ordinal; it is not a validated score or a declaration of deployment suitability.

Platform	Task	Direct ML/DL evidence	Contextual evidence	Validation/deployment gap	Prioritized next study
PSMA	Patient selection/response	Retrospective ML, deep-learning, and automated tumor-burden studies; one interim AI-described abstract.	Dual-tracer imaging, radiomics, and prognostic-association studies.	No validated treatment-effect model or prospective decision-impact test.	Prospective locked-policy decision-impact study.
PSMA	Segmentation/tumor burden	Technical lesion-segmentation and automated tumor-burden reports, including a held-out-center test.	Manual consensus contours and quantitative PET workflow studies.	Activity, absorbed-dose, failure, and correction consequences remain incompletely tested.	Multicenter quantitative and dosimetric validation with human-review endpoints.
PSMA	Dosimetry	Early pretherapy dose prediction, dose-map, registration, and single-time-point workflow reports.	Reference dosimetry, dose-response, and simplified-dosimetry studies.	Limited external reference-based testing; no activity-prescription trial.	Prospective equivalence study followed by a constrained activity-policy trial.
PSMA	Toxicity prediction	Sparse learned-model evidence.	Marrow, renal, salivary, and disease-burden association studies.	No externally validated, calibrated toxicity model linked to a management rule.	Multicenter toxicity-model validation with calibration and prespecified action thresholds.
PSMA	Adaptive therapy	No direct clinical AI-policy evidence identified.	Cycle-by-cycle dosimetry and response-monitoring concepts.	No prospective AI-guided activity or interval adaptation.	Prospective safety-constrained adaptive-policy trial.
SSTR/PRRT	Patient selection/response	Retrospective radiomics-plus-ML and multimodal deep-learning reports.	Conventional radiomics, heterogeneity, and clinical prognostic studies.	Predominantly internal validation; prognosis has not established treatment benefit.	External validation followed by treatment-effect or decision-impact evaluation.
SSTR/PRRT	Segmentation/tumor burden	Human-reviewed organ/lesion segmentation workflow evidence.	Manual contouring and dosimetry workflow references.	Reference contours may depend on corrected AI output; end-to-end benefit is unproven.	Independent-reference multicenter study measuring correction burden and dose impact.
SSTR/PRRT	Dosimetry	Renal/tumor dose prediction and voxel dose-map studies with limited center-held-out testing.	Serial dosimetry, dose-response, and organ-at-risk studies.	Sparse external validation, uncertainty reporting, and threshold-based agreement analysis.	Multicenter reference-dosimetry equivalence and prospective dose-response study.
SSTR/PRRT	Toxicity prediction	No externally validated direct learned-model toxicity study identified.	Marrow and renal dose-toxicity associations.	No externally validated calibrated model linked to a management action.	Prospective toxicity-linked dosimetry cohort with locked prediction and decision rule.
SSTR/PRRT	Adaptive therapy	No direct clinical AI-policy evidence identified.	Dose-guided cycle-adjustment concepts and observational dosimetry.	No prospective decision-impact evaluation.	Safety-constrained prospective adaptive PRRT trial.
Radioiodine	Selection/response and safety/logistics	Retrospective ML response prediction, expert-decision imitation, iodine-avidity classification, and discharge-threshold prediction; one multicenter study used two external cohorts.	Clinical and biochemical risk models and radiation-safety criteria.	Calibration, patient-level independence, and prospective policy effects remain incompletely tested.	Prospective patient-level validation followed by a prespecified decision-impact study.
Radioiodine	Segmentation/monitoring	Deep-learning localization on post-ablation whole-body imaging.	SPECT/CT-informed labels and manual review.	Patient-level independence and external transportability remain incompletely established.	Multicenter patient-separated validation measuring correction burden and management consequences.
Radioiodine	Registration/dosimetry	Single-view ML dosimetry, reduced-time-point neural-network estimation, and internal deformable-registration evidence.	MIRD, Monte Carlo, and serial SPECT/CT reference workflows.	Reference provenance, prespecified agreement margins, and independent dose truth remain limited.	Multicenter patient-level equivalence study against specified reference dosimetry.
⁹⁰Y/TARE	Response/toxicity	Retrospective radiomics-plus-ML reports with mixed internal testing.	Clinical, imaging, and dose-outcome association studies.	Small cohorts, limited calibration, and no prospective management-policy evaluation.	Prospective multicenter locked-model response/toxicity study.
⁹⁰Y/TARE	Planning/dosimetry	Learned attenuation and scatter correction plus technical dose-prediction reports.	Quantitative SPECT/PET, CFD, Monte Carlo, software, and planning workflows.	Technical performance has not established prescription equivalence or decision impact.	Intercenter quantitative equivalence study using a prespecified dosimetric margin.
Emerging RPT	FAP/alpha/Auger and other targets	Sparse platform-specific direct evidence; one exploratory diagnostic-FAPI dose-map report.	Early-phase therapy, radiobiology, and microdosimetry literature.	Insufficient platform-specific external testing and therapeutic endpoints.	Platform-specific prospective technical validation before treatment-policy testing.
Cross-platform	Digital twins/decision support	No direct clinical decision-impact evidence identified.	Frameworks, mechanistic modeling, and computational-oncology concepts.	No locked policy, prospective safety evaluation, or patient-outcome evidence.	Silent prospective evaluation followed by a controlled decision-impact study.

AI, artificial intelligence; CXCR4, C-X-C chemokine receptor 4; PRRT, peptide receptor radionuclide therapy; PSMA, prostate-specific membrane antigen; RPT, radiopharmaceutical therapy; SSTR, somatostatin receptor.

**Table 3:** Selected direct learned-model studies relevant to segmentation, registration, quantitative preprocessing, and dosimetry. Publication type, tracer and input, reference or comparator, validation setting, principal metric, and evidence role are shown. Quantitative and non-AI comparators are tabulated separately in Supplementary File 1.

Study and publication type	Platform, tracer, and input	Task or learned method	Reference or comparator	Validation and reported metric	Evidence role and interpretation
Zhao (83) Peer-reviewed direct-DL original report	PSMA PET lesion characterization	Deep neural network detection and segmentation of PSMA-avid lesions	Manual physician annotations	Retrospective multicenter technical study; 130 training patients and 63 patient-separated test patients pooled across centers; bone-lesion F1 0.99 and lymph-node-lesion F1 0.92; no site-held-out test.	Direct, RPT-motivated technical tumor-burden measurement; no activity, absorbed-dose, or treatment-policy evaluation.
Jackson (80) Peer-reviewed direct-DL original report	PSMA/FDG/Lu-PSMA quantification	Deep learning for automated SUV and molecular tumor volume	Expert consensus segmentation reference.	Retrospective development with a 56-patient holdout described as external; provenance and exact metric denominators remain incompletely reported.	Direct AI quantification; supports automated burden measurement, subject to provenance and metric verification.
Zang (81) Peer-reviewed AI-quantification original report	PSMA tumor burden	Automated PSMA PET tumor-burden metrics	Outcome association.	Retrospective 107-patient cohort; no external test; bootstrap-corrected prognostic C-index 0.71 (95% CI 0.64–0.78).	AI-derived tumor burden with conventional prognostic modeling; prognostic association is not treatment-effect evidence.
Nakaichi (30) Peer-reviewed direct-DL original report	PRRT segmentation/dosimetry	Deep learning segmentation for ^1^⁷⁷Lu-DOTATATE dosimetry	Physicist/physician reference contours and absorbed dose.	Within-cohort comparison; total workflow 47.0 vs 54.3 min (*p* = 0.014); physician approval time not separately reported.	Supervised workflow support; corrected AI output served as the reviewed reference contour.
Chaiwongsa (31) Peer-reviewed AI-segmentation original report	PSMA kidney dosimetry	AI-based versus manual kidney segmentation for single-time-point dosimetry	Manual segmentation and single-time-point dosimetry	Five patients/eight cycles; no dose difference across methods (*p* = 0.964); AI vs. manual DSC 0.898 ± 0.019; no external test.	Small-cohort supervised segmentation/dosimetry workflow evidence only.
Akhavanallaf (111) Peer-reviewed direct-ML original report	SSTR/PRRT tumor dosimetry	Baseline PET-based prediction of tumor absorbed dose	Post-therapy dosimetry reference	Twenty-five patients/90 lesions; nested tenfold cross-validation; random-forest R^2^ 0.64 and MAE 0.73 Gy/GBq; no external cohort.	Direct ML tumor-dose prediction; lesion clustering and absence of external testing limit inference.
Yazdani (93, 94) Peer-reviewed direct-ML/DL original reports	PSMA dosimetry prediction	Radiomics and clinical biomarkers from ⁶⁸Ga-PSMA PET/CT	Monte Carlo-derived or post-therapy absorbed-dose reference.	Twenty-patient retrospective cohorts; LOOCV; kidney RMSE 0.11 Gy/GBq, R^2^ 0.87 and lesion RMSE 1.04 Gy/GBq, R^2^ 0.77; no external test.	Direct ML/DL dosimetry evidence; small cohort and no independent external test.
Mansouri (34) Peer-reviewed direct-DL original report	SSTR/PRRT voxel dosimetry	Hybrid transformer model for ^1^⁷⁷Lu-DOTATATE dose maps	Monte Carlo reference.	Twenty-two patients/50 sessions; patient-grouped fivefold cross-validation; voxel relative absolute error 5.28 ± 1.32 vs. Monte Carlo.	Direct DL dose-map evidence against Monte Carlo; internal technical validation only.
Mansouri (90) Peer-reviewed direct-DL original report	⁹⁰Y-SIRT quantitative preprocessing	Swin UNETR attenuation and scatter correction for ⁹⁹ᵐTc-MAA SPECT	Monte Carlo/scatter-corrected quantitative SPECT and voxel dosimetry.	Retrospective 222-patient dataset; 44-patient blind internal holdout; voxel mean dose error ≤0.003 Gy; no external center.	Direct DL quantitative-preprocessing evidence; the blind test was internal rather than institution-external.
Ha (35) Peer-reviewed direct-DL technical report	Multi-radionuclide dose prediction	Deep-learning voxel dose-kernel framework for beta emitters	Monte Carlo simulation.	Technical kernel/phantom/patient testing; 177Lu test included two patients; kernel R^2^ 0.91 vs. Monte Carlo; no clinical external validation.	Direct DL technical dosimetry evidence; the clinical 177Lu test was very small.
Jalilifar (75) Peer-reviewed direct-ML original report	Radioiodine dosimetry	MLP-based patient-specific ^131^I dosimetry from a single oblique planar view	Monte Carlo reference in external cohort.	23-patient external cohort; organ-specific difference vs. GATE v8.2 Monte Carlo, −14.8% to +11.2%; no overall external MAE/RMSE.	Direct AI dosimetry; method-specific external testing with incompletely described cohort provenance.
Kim (91) Peer-reviewed direct-DL original report	Voxel-wise TIA mapping	Registration and deep learning for TIA prediction	Serial imaging-derived TIA reference	External-center technical test in 14 patients; TIA RMSE about 27% lower for kidney and 33% lower for tumor than Elastix; no dose-decision test.	Direct DL registration/TIA evidence; external technical validation does not establish absorbed-dose or decision validity.
Zhang (38) Peer-reviewed synthetic direct-ML report	Synthetic dose prediction	Semi-supervised dose prediction in targeted radionuclide therapy	Synthetic Monte Carlo-style references.	Synthetic 1,000-phantom study; organ-dose MAPE 9%–11%; no patient or external clinical validation.	Synthetic proof of concept; no clinical validation.
Perret (85) Peer-reviewed original report	PSMA; ⁶⁸Ga-PSMA PET/CT and first-cycle ^1^⁷⁷Lu-PSMA SPECT/CT	nnU-Net lesion segmentation, including PET-guided SPECT models	Four-physician consensus segmentation	Held-out-center test; external Dice 0.76–0.77 (PET) and 0.78 (PET-guided SPECT); unstable PET volume error	Direct AI segmentation evidence; activity, absorbed dose, and treatment decisions not evaluated
Liu (96) Peer-reviewed original report	Radioiodine/DTC; serial quantitative ^131^I SPECT/CT	Unsupervised deformable registration with Swin-transformer encoders	Elastix and UTSRMorph registration comparators	58-patient pretraining set; 12-patient serial set; five explicitly held-out evaluation patients; no independent deformation/dose truth	Direct AI registration evidence; improved technical consistency, not proven absorbed-dose accuracy
Izadi-Yazdi (97) Peer-reviewed original report	FAPI; ⁶⁸Ga-FAPI-46 and ^1^⁸F-FDG PET/CT in 22 patients	Swin UNETR dose-rate-map prediction	Dose-voxel kernel, full Monte Carlo, local energy deposition, and OLINDA/EXM	Internal cross-validation; inconsistent stated map reference and unclear patient-level fold isolation	Exploratory direct AI evidence using diagnostic ⁶⁸Ga maps; not therapeutic-dose validation
Georgiou (95) Peer-reviewed direct-DL original report	Radioiodine/DTC; 4-, 24-, and 48-hour imaging and blood data	Feed-forward neural-network prediction of MIRD maximum permissible activity	Full five-time-point MIRD calculation	70 training and 13 previously unused test patients; paired difference *p* = 0.351 (95% CI, −21.05 to 8.08 mCi); no equivalence margin	Internal proof of concept; a nonsignificant difference does not establish equivalence, and clinical prescription or decision impact was not tested
Kavitha (73) Peer-reviewed direct-DL original report	Radioiodine/PTC; post-ablation planar whole-body scans	Multilayer fully connected network for metastatic lymph-node localization	SPECT/CT-informed labels and physician comparison	Retrospective 230-patient cohort plus 30-scan same-center pilot; test F1 82.3%–84.4%; pilot Dice 73%	Supervised localization evidence; patient-level independence incompletely documented and no downstream decision impact

AI, artificial intelligence; CT, computed tomography; DTC, differentiated thyroid carcinoma; MLP, multilayer perceptron; PET, positron emission tomography; PRRT, peptide receptor radionuclide therapy; PSMA, prostate-specific membrane antigen; SPECT, single-photon-emission computed tomography; TIA, time-integrated activity.

**Table 4:** Selected data-driven studies of treatment stratification, prognostication, response assessment, and safety/logistics in radiopharmaceutical therapy.

Study and publication type	Platform	AI/data approach	Endpoint	Study design and validation	Interpretation and limitation
Luo (41) Peer-reviewed direct-ML original report	Radioiodine/DTC	LightGBM with SHAP; 950 patients; clinical, laboratory, administered activity, and post-therapy scan variables.	Non-excellent response after initial ^131^I therapy	This was a retrospective, single-center study.	Post-treatment response stratification, not pretreatment selection.
Baur (40) Peer-reviewed direct-DL original report	SSTR/PRRT	Multimodal deep learning using SSTR PET/CT and laboratory variables; 116 patients	Short versus long PFS after [^1^⁷⁷Lu]Lu-DOTATOC PRRT	Retrospective, single-center, repeated three-fold cross-validation.	Developmental PFS prediction; no external validation was performed.
Singh (63) Peer-reviewed conventional-model context report	SSTR/PRRT	Clinicopathologic variables with [^1^⁸F]FDG PET positivity in a nomogram	Overall survival probability in metastatic pancreatic NEN treated with PRRT	Retrospective internal validation	Prognostic risk stratification; no prospective treatment selection evidence.
Murthy (60) Peer-reviewed AI-described original report	PSMA	AI-assisted lesion tracking on PSMA PET	Prognosis after [^1^⁷⁷Lu]Lu-PSMA-617 therapy	Retrospective single-center cohort study	End-of-treatment lesion tracking, not pretreatment selection.
Laudicella (62) Peer-reviewed radiomics/conventional-model context report	SSTR/PRRT	SSTR PET/CT radiomics and clinical features; 38 patients/324 lesions in the ‘Theragnomics’ study.	Response prediction in GEP-NETs undergoing PRRT	Retrospective internal k-fold lesion-level modeling with no external validation.	Developmental evidence: Within-patient lesion clustering may inflate precision.
Ghazizadeh (51) Peer-reviewed radiomics-plus-ML original report	SSTR/PRRT	[⁹⁹ᵐTc]Tc-octreotide SPECT radiomics and clinical variables in 93 patients.	mRECIST response after PRRT	Retrospective multicenter pool with internal testing; no held-out sites.	Developmental response prediction
Bülbül (59) Peer-reviewed direct-ML original report	PSMA	Pretreatment PSMA PET texture features and ML: 9 patients/174 lesions.	Lesion-level progression after two cycles of ^1^⁷⁷Lu-PSMA therapy	Retrospective single-center lesion-level analysis	Substantial small-sample and within-patient clustering limitations were observed.
Rios-Sanchez (43) Peer-reviewed radiomics-plus-ML original report	PSMA	Automated total tumor volume and LASSO-based ML	PSA50 response after ^1^⁷⁷Lu-PSMA therapy	Single-center fivefold cross-validation	No independent external validation was performed.
Sa (74) Peer-reviewed direct-ML original report	Radioiodine/DTC	Routine pre-treatment clinical variables with random forest models	Effective response and biochemical remission after ^131^I therapy	Retrospective single-institution internal validation	Risk stratification, not activity prescription.
Pan (39) Peer-reviewed conventional-model/radiomics context report	PSMA	Dual-tracer PSMA/FDG radiomics with a conventional stepwise logistic regression nomogram	Probability of PSMA-negative/FDG-positive disease and downstream response risk.	Retrospective multicenter development; separate cohort tested prognostic association.	The primary classification AUC was not externally validated.
Djaileb (57) Contextual meeting abstract; prospective enrollment; interim report; method family not reported	PSMA	AI-based SelectPSMA stratification, interim Grenoble cohort, 60 patients.	PSA50 and progression-free survival after [^1^⁷⁷Lu]Lu-PSMA therapy.	Prospective enrollment at two institutions; interim analysis from one institutional cohort; meeting abstract.	Preliminary prognostic evidence; not completed multicenter validation or decision impact analysis.
Ince (42) Peer-reviewed radiomics-plus-ML original report	⁹⁰Y/TARE	Clinical and MRI radiomic features with standard ML	Short-term EASL response after ⁹⁰Y radioembolization	Retrospective single-center nested internal cross-validation	Feasibility; small non-responder group.
Mosconi (77) Peer-reviewed radiomics/context report	⁹⁰Y/TARE	Pretreatment CT texture analysis	Objective response and PFS in intrahepatic cholangiocarcinoma after ⁹⁰Y radioembolization	Retrospective two-center exploratory cohort; no derivation-validation split or independent validation was performed.	Secondary-platform quantitative context
Topcuoglu (78) Peer-reviewed radiomics-plus-ML original report	⁹⁰Y/TARE	[^1^⁸F]FDG PET radiomics with random forest and clinical-radiomic models	Local response after TARE for colorectal liver metastases	Single-center hold-out test; test *n* = 30.	Feasibility: No external validation.
Gafita (58) Contextual peer-reviewed AI-described report; method family not reported	PSMA	Proprietary SelectPSMA classification from baseline PSMA PET/CT; 76 patients, with output available for 71.	PSA response, PSA progression-free survival, and overall survival after ^1^⁷⁷Lu-PSMA-617.	Retrospective single-center cohort; reportedly not used for technology development/validation; development-site provenance unclear.	Prognostic association only; no prospective locked-model validation or decision-impact evaluation.
Popovic (65) Peer-reviewed direct-ML original report	Radioiodine/DTC	Artificial neural network, naïve Bayes, and group method of data handling; reported cohort *n* = 210.	Expert-imitated recommendation: no radioiodine or 1.85, 3.7, or 5.55 GBq.	Retrospective single-center internal split; reported 150 training plus 70 test patients conflicts with total *n* = 210.	ANN accuracy 95.71%, weighted κ=0.96 (95% CI 0.91–1.00); expert-decision imitation, not outcome or treatment-benefit validation.
Bülbül and Nak (66) Peer-reviewed direct-ML original report	Radioiodine/DTC	Nine classifiers; 151 patients; 80/20 internal split with tenfold cross-validation in training.	Excellent response 6–24 months after radioiodine; inputs included activity and post-ablation whole-body imaging.	Single-center internal testing; Table 2: extreme-gradient-boosting AUC 0.865; gradient-boosting accuracy 81%.	Post-treatment response prediction, not pretreatment activity selection; inconsistent cohort/event and AUC reporting; no external validation.
Gao (67) Peer-reviewed direct-DL original report	Radioiodine/DTC lung metastases	SE V-Net segmentation and SE-Net iodine-avidity classification; 496-patient development cohort and 24-patient external cohort.	Iodine avidity on post-treatment SPECT following pretreatment chest CT.	Lesion-level development split; external AUC 0.713 (95% CI 0.613–0.813) in 123 lesions from two hospitals.	External degradation and possible within-patient leakage/clustering; no prospective treatment-policy or benefit evaluation.
Song (68) Peer-reviewed direct-DL original report	Radioiodine/DTC lung metastases	ResNeSt50 prediction of ^131^I uptake from pretreatment chest CT; 404 patients across three hospitals.	Iodine avidity on post-therapy whole-body imaging.	Temporal internal validation plus two institution-external cohorts; external AUCs approximately 0.72–0.73.	Direct pretreatment stratification evidence; minor internal numerical inconsistencies and no prospective model-guided treatment or benefit evaluation.
Zhang (71) Peer-reviewed direct-ML original report	Radioiodine/DTC	Support-vector machine using clinical and measured residual-activity features in 1,044 treated patients.	Whether the regulatory residual-activity discharge threshold would be met 48 h after ^131^I.	Retrospective single-center internal evaluation; accuracy 88.04% and precision 96.89%.	Radiation-safety/logistics decision support; not a treatment-efficacy, activity-selection, or adaptive-policy study.

AI, artificial intelligence; AUC, area under the receiver operating characteristic curve; CT, computed tomography; DTC, differentiated thyroid carcinoma; EASL, European Association for the Study of the Liver; FDG, fluorodeoxyglucose; GEP-NET, gastroenteropancreatic neuroendocrine tumor; LASSO, least absolute shrinkage and selection operator; LightGBM, Light Gradient Boosting Machine; ML, machine learning; MRI, magnetic resonance imaging; mRECIST, modified Response Evaluation Criteria in Solid Tumors; NEN, neuroendocrine neoplasm; PFS, progression-free survival; PRRT, peptide receptor radionuclide therapy; PSA50, at least 50% decline in prostate-specific antigen; PSMA, prostate-specific membrane antigen; SHAP, Shapley additive explanations; SSTR, somatostatin receptor; TARE, transarterial radioembolization.

### 5.1 Decision relevance and endpoint selection

We identified four inferential targets. Prognostic models estimate the risk under observed care, response models estimate an endpoint after treatment, treatment-effect models estimate outcomes under specified alternatives, and treatment policies map patient states to actions. Only the latter two can directly justify choosing or withholding treatment. Target trial emulation, causal machine learning, individualized treatment effect estimation, and other counterfactual analyses remain hypothesis-generating unless timing, eligibility, confounding, positivity, censoring, overlap, calibration, and decision impact are addressed, and they still require prospective evaluation of a prespecified management rule (56).

### 5.2 PSMA-targeted radioligand therapy

In PSMA-targeted prostate cancer therapy, a clinically relevant application is the identification of a biological mismatch before RLT. Pan et al. developed a dual-tracer radiomics nomogram to estimate PSMA-negative/[^1^⁸F]fluorodeoxyglucose (FDG)-positive disease. In the development cohort, the model achieved an area under the receiver operating characteristic curve (AUC) of 0.83, compared with 0.78 for the clinical model and 0.67 for the previously published Renji model. Decision analysis estimated that a 5% threshold could avoid 21% of FDG PET/CT examinations while missing 2% of PSMA-negative/FDG-positive cases. A separate Nanjing cohort tested the associations between model-predicted risk and outcomes after [^1^⁷⁷Lu]Lu-PSMA-617, but it did not independently validate the primary classification AUC. Therefore, we regarded this as an external prognostic association rather than an external validation of the selection model (39).

Xue et al. reported peer-reviewed dual-tracer PET/CT biomarkers associated with outcomes after PSMA RLT. The findings support concordance-aware risk stratification, but the small external cohort limits definitive performance claims (50).

One emerging prospective dataset was reported in a 2025 JCO meeting abstract from the SelectPSMA study. Although patients were prospectively enrolled at two institutions, the reported interim analysis included only the 60-patient Grenoble cohort and evaluated associations with PSA50 and PSA progression-free survival; it did not test an AI-guided management rule. Therefore, it is preliminary contextual prospective prognostic evidence, not a completed multicenter validation or decision-impact evidence (57).

A related peer-reviewed report by Gafita et al. retrospectively evaluated 76 patients treated at one center after regulatory approval; SelectPSMA output was available for 71. The cohort was reported not to have contributed to the development or validation of the technology, but the proprietary model family and development-site provenance were not described. We therefore classify it as an AI-described original human report outside the strict direct-ML/DL denominator, not as institution-external validation or evidence of decision impact. This cohort and publication are distinct from the later Djaileb et al. prospective meeting abstract (57, 58).

Bülbül et al. used pretreatment PSMA PET texture features and machine-learning classifiers to predict lesion-level progression after two cycles of [^1^⁷⁷Lu]Lu-PSMA therapy. The cohort contained 9 patients and 174 lesions, so patient-level clustering, small-sample instability, and absence of external validation are important limitations (59). Murthy et al. evaluated AI-assisted lesion tracking on baseline and end-of-treatment PSMA PET in a small retrospective single-center cohort; this is longitudinal response/prognostic evidence rather than pretreatment selection evidence (60). Neither study supports treatment withholding or activity adaptation without prospective evaluation.

Gutsche et al. provide peer-reviewed multicenter PSMA PET radiomics evidence for [^1^⁷⁷Lu]Lu-PSMA-617 response assessment (61). Conference abstracts were analyzed separately, excluded from denominators based on the 73 full journal reports, and not treated as methodologically equivalent to peer-reviewed full reports.

### 5.3 SSTR-targeted PRRT

Baur et al. evaluated multimodal deep learning in 116 patients with metastatic neuroendocrine tumors who underwent [^1^⁷⁷Lu]Lu-DOTATOC PRRT. The PET/CT/laboratory fusion model achieved an AUC of 0.72 ± 0.01 with five-times-repeated threefold cross-validation. This supports complementary imaging and laboratory information, but remains retrospective, single-center, internally validated evidence without external validation (40).

Laudicella et al. reported the ‘Theragnomics’ concept using SSTR PET/CT radiomics and conventional statistical modeling to study response in gastroenteropancreatic neuroendocrine tumors undergoing PRRT. We retained this study as radiomics and quantitative-prediction context rather than in the direct AI denominator. Its lesion-level analysis remains developmental because within-patient clustering, cohort size, and lack of external validation limit inference (62).

Ghazizadeh et al. used pretreatment [⁹⁹ᵐTc]Tc-octreotide SPECT radiomics and clinical variables in 93 patients to predict modified Response Evaluation Criteria in Solid Tumors (mRECIST) response after PRRT; the random forest model reported an AUC of approximately 0.89 in an internal test setting (51). Singh et al. trained a random survival forest to predict overall survival in 447 patients with metastatic pancreatic neuroendocrine neoplasms treated with PRRT, using a random 80/20 single-center split; the reported C-index was 0.86 in development and 0.82 in the 90-patient internal test set. Variables selected by the random survival forest were subsequently entered into a Cox proportional hazards model to construct the displayed PANEN nomogram. Therefore, we classified the report as hybrid direct learned-model prognostic evidence while distinguishing the Cox-based nomogram from the random survival forest and noting the absence of external validation or prospective treatment-policy evaluation (63). These studies support developmental risk stratification but do not validate AI-guided treatment selection.

Pretherapy SSTR heterogeneity is also a prognostic factor. Werner et al. reported an association between SSTR-based heterogeneity and OS in pancreatic neuroendocrine tumor patients undergoing PRRT; however, the study was not an AI-guided treatment-selection system (64).

### 5.4 Radioiodine therapy

Radioiodine therapy presents a different decision context. Luo et al. developed a light gradient boosting machine (LightGBM) model interpreted with Shapley additive explanations (SHAP) in 950 treatment-naive patients with differentiated thyroid carcinoma (DTC), reporting a test-set AUC of 0.896 versus 0.842 for logistic regression. Because the model incorporated administered activity and post-therapy scan variables, it informs post-treatment response stratification rather than pretreatment selection. This was a retrospective, single-center internal-split study (41).

Popovic et al. compared an artificial neural network, naïve Bayes classifier, and group method of data handling for recommending no postoperative radioiodine or 1.85, 3.7, or 5.55 GBq in differentiated thyroid carcinoma (DTC). Against a single experienced physician's reassessed decision, the neural network achieved 95.71% test accuracy and weighted κ=0.96 (95% CI 0.91–1.00). The report describes 210 patients but a 150-patient training set plus a 70-patient test set, which is an unresolved numerical inconsistency. Because the target was expert decision imitation rather than recurrence, toxicity, or treatment benefit, and testing was single-center and internal, this study supports educational decision assistance but not automated activity selection (65).

Bülbül and Nak retrospectively evaluated nine machine-learning classifiers for predicting an excellent response 6–24 months after radioiodine treatment in 151 patients with differentiated thyroid carcinoma without distant metastasis. With an 80/20 internal split and tenfold cross-validation in the training portion, the source report gives a test AUC of 0.865 for extreme gradient boosting and 81% accuracy for gradient boosting. Inputs included administered radioiodine activity and the post-ablation whole-body scan; therefore, the model is a post-treatment response predictor rather than a validated pretreatment selection or activity-optimization tool. The report contains inconsistent cohort/event counts and reports the leading AUC as 0.871 in the abstract and narrative versus 0.865 in its tabulated results; no external validation was performed (66).

Gao et al. trained deep networks to segment and classify 1,962 pretreatment lung metastases in 496 patients as iodine-avid or non-iodine-avid and tested the classifier in 123 lesions from 24 patients at two other hospitals. The reported AUC decreased from 0.879 (95% CI 0.852–0.906) internally to 0.713 (95% CI 0.613–0.813) externally. Because the development split was lesion-level despite multiple lesions per patient, within-patient leakage or clustering cannot be excluded; the external patient sample was small, contours required radiologist review, and no prospective treatment-policy or treatment-benefit evaluation was performed (67).

The final search in July 2026 added four eligible direct radioiodine reports. Song et al. trained a deep convolutional network in 404 patients and used temporal internal validation plus two institution-external cohorts to predict pretreatment ^131^I uptake in lung metastases; reported external AUCs were approximately 0.72–0.73, although values differed slightly between the source article's narrative and its tabulated results (68). Lubin et al. evaluated 107 patients, including 46 initial treatment failures; the report described the model inconsistently but identified a random forest as the final classifier and did not report discrimination or external validation (69). Su et al. evaluated several learned classifiers before radioiodine imaging; qualifying direct-model results included SVM AUC 0.93 and random-forest AUC 0.92, whereas the authors’ preferred logistic regression model remains a conventional statistical context under our definition (70). Zhang et al. used an SVM in 1,044 patients to predict whether the residual-activity discharge criterion would be met at 48 h (accuracy 88.04%, precision 96.89%); this is radiation safety and logistics support, not treatment efficacy or adaptive prescription evidence (71). A systematic review published before the search endpoint was retained as a contextual synthesis and did not enter the direct-report denominator (72).

Kavitha et al. developed a multilayer fully connected network to localize iodine-avid metastatic lymph nodes on post-ablation planar whole-body scans in a retrospective 230-patient cohort, using SPECT/CT-informed labels and an additional 30-scan pilot. Test F1 scores were 82.3%–84.4%, and the pilot metastatic-node Dice score was 73%. Patch- or region-level analysis, incompletely documented patient-level independence, one-center acquisition, and absence of downstream management testing limit the result to supervised localization and monitoring support (73).

Sa et al. used routine pretreatment variables to predict effective response to ^131^I therapy and later biochemical remission. The random forest models reported test-set AUCs of 0.896 and 0.857, respectively, in a retrospective single-institution dataset. These results support risk stratification and counseling rather than patient-specific activity prescription or treatment withholding (74).

Therefore, retained radioiodine evidence centers on oncologic DTC avidity or response stratification, localization, radiation-safety logistics, and dosimetry, rather than non-oncologic thyroid disease (7, 41, 65–76).

### 5.5 ⁹⁰Y radioembolization

Ince et al. used preprocedural clinical features and arterial-phase MRI radiomics to predict the three-month European Association for the Study of the Liver response after ⁹⁰Y transarterial radioembolization (TARE) in HCC. Nested internal cross-validation yielded AUCs up to 0.94, but the single-institution dataset and small non-responder group limited generalizability (42).

Mosconi et al. associated pretreatment CT texture features with response and PFS after ⁹⁰Y radioembolization for intrahepatic cholangiocarcinoma (77). Topcuoglu et al. reported a test-set AUC of 0.90 for an FDG PET radiomics random forest model in colorectal liver metastases; the single-center test set contained 30 patients (78). Öcal et al. provided additional radiomics evidence of colorectal liver metastases (79). These studies support feasibility rather than implementation.

Radioembolization-induced liver disease (REILD) is a distinct pretreatment toxicity. Rivera et al. developed machine-learning classifiers for REILD risk and bilirubin threshold refinement; this evidence is considered hepatic toxicity rather than generic response selection (52).

## 6 Automated segmentation and tumor-burden quantification for theranostics

In RPT, segmentation is a quantitative measurement step rather than a display function: contours determine tumor burden, organ activity, time-activity curves, and sometimes dose geometry. Dice, Hausdorff distance, recall, and precision remain useful development metrics, but validation should also test lesion size and physiologic uptake failures, activity recovery, time-integrated activity, absorbed-dose consequences, correction burden, and failed cases. The acceptable error depends on the structure and action; a small boundary error may be negligible in a large homogeneous organ but important in a small lesion, salivary gland, renal subregion, or marrow compartment. Manual contours are imperfect references; therefore, reference construction and inter-reader variability must be reported. The evidence is therefore interpreted as supervised lesion inventory, organ delineation, tumor-burden reporting, and dosimetry preparation unless a treatment consequence was directly tested (29–33, 80–83).

### 6.1 PSMA lesion segmentation and tumor-burden quantification

PSMA imaging has a substantial mapped literature on lesion delineation and whole-body tumor burden, including total tumor volume, uptake, lesion counts, heterogeneity, and spatial distribution. These outputs may support supervised lesion inventory, reporting, response assessment, or dosimetry preparation, but most studies are retrospective, and none establishes that AI-defined volume alone should determine activity, escalation, or cessation. Intraprostatic segmentation in primary prostate cancer remains a technical context rather than direct RLT-planning evidence. Predictive use requires a clinically meaningful endpoint, transparent cohort and validation design, and comparison with simpler clinical or imaging criteria (43, 54, 80–84).

Perret et al. retrospectively evaluated nnU-Net lesion segmentation on paired ⁶⁸Ga-PSMA PET/CT and first-cycle ^1^⁷⁷Lu-PSMA SPECT/CT in 73 patients, training at one center and testing at a second. External Dice scores were 0.76–0.77 for PET and 0.78 for PET-guided SPECT segmentation, but PET volume errors were highly unstable, and activity or absorbed-dose consequences were not tested. Therefore, this study is a genuine held-out-center technical validation, not evidence that automated contours improve dosimetry or treatment decisions (85).

### 6.2 Organ-at-risk segmentation and normal-tissue quantification

Organ-at-risk segmentation is central to personalized dosimetry because absorbed doses to the kidneys, marrow, liver, and lungs can constrain treatment intensity, depending on the radiopharmaceutical and disease setting. Automated organ delineation can reduce the workload, improve consistency, and enable multi-organ reporting; however, organ segmentation must be evaluated against the absorbed-dose consequences, not only against an anatomic contour. This is especially important when the activity is heterogeneous within an organ or when the relevant biological target is a suborgan compartment rather than a whole-organ volume (29–33).

Among the identified evidence, salivary and lacrimal toxicity, particularly with PSMA-targeted and alpha-emitting therapies, was not represented by a validated AI prediction model. Development is constrained by small gland size, anatomical variability, physiologic uptake and spill-in, inconsistent contouring and dosimetry methods, heterogeneous toxicity endpoints, and sparse longitudinal datasets linking gland dose to patient-reported and clinician-graded outcomes. Therefore, small-gland segmentation, symptom prediction, and dose-toxicity modeling should be treated as separate future tasks rather than established applications. Marrow segmentation and toxicity modeling are also limited by spatially distributed marrow, lesion-confounded uptake, and heterogeneous blood-based surrogates (14, 15, 45, 53, 54).

### 6.3 SSTR/PRRT and radioiodine applications

Within SSTR/PRRT, segmentation evidence is tied mainly to the supervised dosimetry workflow. In 23 patients, Nakaichi et al. reported a median total time of 47.0 min for reconstruction, AI processing and contour correction, and dose calculation versus 54.3 min manually (*p* = 0.014); physician approval time was not separated, and the reference was the staff-corrected AI contour. This result supports modest supervised acceleration, not autonomous segmentation or an independent reference. In the mapped radioiodine literature, segmentation evidence for treatment planning was limited; studies instead addressed response prediction or activity modeling, while quantitative imaging, localization, kinetics, and avidity remain necessary for ^131^I dosimetry (30, 41, 74–76).

### 6.4 ⁹⁰Y radioembolization and quantitative preprocessing

In ⁹⁰Y radioembolization, segmentation intersects with a different set of quantitative problems: liver and tumor compartment definition, lung shunt estimation, treatment planning software, microsphere distribution, and post-treatment PET or SPECT dosimetry. AI may contribute through tumor or liver segmentation, radiomics-based response prediction, or dose-map prediction; however, accurate quantitative imaging and compartment definition remain prerequisites. Reconstruction choices, partial-volume effects, registration, and software assumptions can materially affect the reported absorbed dose; therefore, image quality improvement should not be equated with preserved quantitative fidelity unless dosimetric consequences are tested (42, 52, 79, 86–90).

## 7 AI-assisted personalized dosimetry

Personalized dosimetry links quantitative imaging to treatment adaptation. A specified reference combines calibrated activity measurements, volumes of interest, kinetic modeling, and an explicit MIRD, voxel-S-value, dose-kernel, or Monte Carlo formalism. AI may simplify segmentation, registration, reconstruction, sampling, or calculation; however, the relevant endpoint remains agreement in activity and absorbed dose, with uncertainty, for the intended organ, lesion, and decision. A model trained on Monte Carlo maps answers a different question from one trained on organ-level MIRD estimates or a software-specific workflow; neither establishes that AI-guided prescription improves outcomes (8–11, 32–37, 91).

### 7.1 Quantitative imaging and preprocessing are not peripheral details

AI-assisted dosimetry depends on quantitative image fidelity before any model is applied. Reconstruction and preprocessing can alter the activity concentration and dose-volume quantities, especially for high-gradient lesions or small organs at risk. In ⁹⁰Y radioembolization, reconstruction-dependent variability illustrates that not all dose metrics are equally robust; some voxel-derived summary measures were relatively stable across reconstruction settings, whereas maximum-dose metrics were more sensitive to the reconstruction choice. This supports a broader principle for all RPT platforms: image-quality improvement is acceptable only if activity quantification and the downstream absorbed-dose quantities needed for treatment decisions are preserved (86–90, 92).

### 7.2 Reduced-time-point and pretherapy dose prediction

As a quantitative prediction context, Peterson et al. used internally validated regression models to link pretherapy [⁶⁸Ga]Ga-DOTATATE renal uptake with the first-cycle [^1^⁷⁷Lu]Lu-DOTATATE renal absorbed dose. The PET-only mean absolute percentage error (MAPE) was 18.0% and 17.3% when the estimated glomerular filtration rate was added. The leave-one-out analysis is clinically relevant but remains internally validated (37).

Akhavanallaf et al. used a random forest to predict the lesion absorbed dose in 25 patients (90 lesions), reporting a nested tenfold cross-validated R^2^ of 0.64 and mean absolute error of 0.73 Gy/GBq; no external cohort was evaluated (93). In a separate five-center study, Akhavanallaf et al. used a mixed-effects model with leave-one-center-out validation and reported an external mean relative absolute error of 28%. The multicenter design is stronger than a random internal split, but neither report established prospective activity adjustment or replacement of post-therapy measurements (36).

For PSMA-targeted RLT, machine learning models using pre-therapy ⁶⁸Ga-PSMA PET/CT radiomic and clinical features have been used to predict the kidney and lesion absorbed dose after [^1^⁷⁷Lu]Lu-PSMA-617. Yazdani et al.'s treatment planning studies provide examples of pre-therapy organ and lesion dose prediction against Monte Carlo-derived or post-therapy dosimetry references. Interpretation is limited by small or single-center cohorts, internal or leave-one-out validation, dependence on the reference dosimetry pipeline, and the absence of demonstrated decision-impact use (94, 95).

Jalilifar et al. trained a multilayer-perceptron model for patient-specific ^131^I planar dosimetry from a single oblique view and compared estimates with GATE v8.2 Monte Carlo dosimetry. In the 23-patient external cohort, reported organ-specific percentage differences ranged from −14.8% to +11.2%. The provenance of the external cohort relative to model development was not fully described, and no single overall external MAE or RMSE was reported; therefore, the result is method-specific external testing rather than broad validation (75).

Georgiou et al. trained a feed-forward neural network in 70 patients to estimate the MIRD maximum permissible ^131^I activity from 4 to , 24-, and 48-hour imaging and blood data, then compared predictions with full five-time-point MIRD calculations in 13 previously unused patients. The paired mean difference was not statistically significant (*p* = 0.351; 95% CI −21.05–8.08 mCi), but a nonsignificant difference does not establish equivalence, no clinical margin or limits-of-agreement analysis was prespecified, and one of 13 tabled predictions differed from MIRD by more than 10%. The small single-center test supports proof of concept for reduced-time-point estimation, but does not replace reference dosimetry or validated activity prescription (96).

### 7.3 Bone marrow dosimetry and hematologic linkage

Bone marrow dosimetry and hematologic toxicity are clinically important but technically challenging. The SPECT/CT-derived red marrow absorbed dose has been associated with toxicity after [^1^⁷⁷Lu]Lu-DOTATATE, and PSMA PET tumor-burden features have been examined in relation to hematologic toxicity after [^1^⁷⁷Lu]Lu-PSMA-617 (53, 54). No prospectively validated AI rule for activity modification has been developed based on these associations.

### 7.4 Lesion-level and voxel-level absorbed-dose estimation

Lesion- and voxel-level dosimetry are attractive because the RPT response is spatially heterogeneous. The mean organ dose can obscure both underdosed lesions and spatially concentrated normal-tissue exposure, although clinically validated thresholds for voxel-level metrics are limited. AI models can support this granular view by predicting the absorbed dose of the lesion, generating voxel-wise dose maps, or approximating Monte Carlo simulations. The voxel-level output should not be interpreted as inherently more clinically useful than the organ-level output unless a dose-response or dose-toxicity relationship exists at the same spatial scale (34, 35, 38, 86–88, 94, 95).

Technical examples include transformer-based [^1^⁷⁷Lu]Lu-DOTATATE voxel-dose prediction, radiodosiomics, and Swin UNETR models for [^1^⁷⁷Lu]Lu-PSMA-617 absorbed-dose prediction, and deep-learning-guided attenuation and scatter correction for [⁹⁹ᵐTc]Tc-MAA SPECT toward ⁹⁰Y-SIRT quantification (34, 90, 94, 95). These models address different radionuclides, inputs, and reference endpoints and should not be treated as interchangeable models.

Liu et al. developed an unsupervised deformable SPECT/CT registration framework for voxel-level ^131^I dosimetry using inter-patient pretraining in 58 patients and serial scans from 12 additional patients. Five patient-held-out evaluation sessions were reported. Relative to Elastix, tumor time-activity-curve fitting RMSE was 11.7% lower; however, no external site or independent deformation or *in-vivo* absorbed-dose ground truth was available. The result indicates technical consistency, not proven dosimetric accuracy or a prescription rule (97).

Izadi-Yazdi et al. used Swin UNETR to predict computational ⁶⁸Ga-FAPI-46 PET/CT dose-rate maps in 22 patients. In internal fivefold cross-validation, the authors reported R^2^ of 0.960 and a gamma pass rate of 98.71%. The report inconsistently identifies dose-voxel-kernel versus full Monte Carlo maps as the reference, and patient-level fold isolation was not explicit. The study also used a diagnostic ⁶⁸Ga tracer rather than a therapeutic FAPI radionuclide. We retained it as exploratory direct technical evidence because the learned voxel-map task could inform future therapeutic dosimetry; it is not validation for a therapeutic tracer, treatment plan, or activity-selection rule (98).

### 7.5 Deep learning surrogates for Monte Carlo and dose-kernel methods

Ha et al. described a multi-radionuclide deep learning framework for beta-emitter dose prediction, with more substantial validation in ⁹⁰Y than in the two-patient [^1^⁷⁷Lu]Lu-DOTATATE sample (35). Mansouri et al. used AI-guided SPECT preprocessing for ⁹⁰Y-SIRT quantitative analysis (90). Both studies provide technical evidence rather than treatment-decision validation.

The most defensible language is comparative rather than prescriptive: AI may approximate or accelerate Monte Carlo-style dosimetry under the conditions in which it was trained and validated; however, it does not yet constitute a generalizable replacement for specified reference dosimetry across tracers, scanners, reconstruction protocols, organ systems, and treatment settings. This is particularly important for cross-radionuclide or zero-shot dose-prediction studies, which are methodologically important but remain technical evidence until externally and prospectively validated (35, 94).

### 7.6 Clinical interpretation: from faster dose calculation to treatment-changing dosimetry

Clinical use of AI-assisted dosimetry requires linked evidence: preservation of activity and absorbed-dose accuracy for the intended organ or lesion; uncertainty and out-of-domain detection; an actionable dose-response or dose-toxicity relationship; a prespecified clinical action; and prospective comparison with conventional care. The current map supports supervised segmentation, faster calculation, reduced imaging burden, standardized reporting, and triage for more detailed dosimetry, but not autonomous or response-adaptive activity prescription (8, 11, 30, 31, 34–37, 53, 55, 99).

Correlation is not evidence of agreement or interchangeability. Method-comparison studies should report Bland–Altman bias and limits of agreement, repeatability coefficients where repeated measurements are available, calibration intercepts and slopes, empirical interval-coverage probabilities, and propagated uncertainty; clustered organs, lesions, scans, or cycles must be handled at the patient level. Organ-specific equivalence or noninferiority margins should be justified for the intended action. A margin acceptable for workflow triage may be unsafe near a renal, marrow, pulmonary, or hepatic constraint. Therefore, replacement of a reference method requires prespecified agreement testing and reporting of clinically consequential decision-threshold crossing errors, not only correlation or average error (100).

## 8 Toxicity prediction, response-adaptive therapy, and theranostic digital twins

In the mapped set, toxicity and adaptive treatment evidence consisted of retrospective prediction studies, contextual dose-response or dose-toxicity reports, technical workflows, and conceptual models; no prospective AI-guided treatment-policy trial was identified (20–22, 52–55, 101).

### 8.1 Toxicity endpoints must be clinically specific and dose linked

Endpoints must remain specific because hematologic, renal, salivary, lacrimal, hepatic, pulmonary, and fatigue outcomes differ in terms of mechanism, timing, competing risks, and relevant exposure. Imaging radiomics supports risk stratification but is not dose-toxicity modeling without quantitative exposure, whereas dosimetry alone may omit baseline function, prior therapy, marrow reserve, tumor burden, and non-radiation causes. Models intended to guide cycles therefore require clinically meaningful endpoints and combined exposure and vulnerability information (52–55, 101).

### 8.2 Hematologic and bone marrow toxicity prediction

Hematologic toxicity can limit ^1^⁷⁷Lu-PSMA and PRRT continuation, even when target expression remains favorable. AI may support marrow-risk assessment through automated marrow or blood-pool segmentation, skeletal tumor-burden quantification, and longitudinal models combining blood counts, renal function, prior therapy, cumulative activity, tumor burden, and post-therapy dosimetry. Current evidence does not establish a prospectively validated AI rule for activity reduction, delay, or discontinuation (53, 54).

Currently, marrow-related AI provides risk support rather than treatment direction. Associations between absorbed dose, disease burden, blood count decline, and response do not indicate that an AI-guided intervention improves outcomes (53, 54).

### 8.3 Renal, salivary, lacrimal, and other organ-at-risk toxicity

Kidney dosimetry illustrates a possible connection between workflow automation and the prevention of toxicity. AI-supported segmentation and pretherapy dose prediction may reduce workload and support earlier risk estimation; however, available studies remain dependent on local quantitative workflows and do not establish a universal action threshold (36, 37, 101).

Among the identified evidence, salivary and lacrimal toxicity, particularly with PSMA-targeted and alpha-emitting therapies, was not represented by a validated AI prediction model. Therefore, small gland segmentation, symptom prediction, and dose-toxicity modeling should be treated as separate future tasks rather than established applications (14, 15, 45).

### 8.4 Hepatic and pulmonary toxicity in ⁹⁰Y radioembolization

⁹⁰Y radioembolization differs from systemic ^1^⁷⁷Lu therapy because the planning centers on lung shunt, nontumoral liver dose, functional liver reserve, treatment volume, microsphere distribution, and REILD risk. AI and computational methods have been investigated for segmentation, quantitative ⁹⁹ᵐTc-MAA SPECT/CT or ⁹⁰Y PET analysis, microsphere modeling, and liver toxicity or response prediction. These applications remain platform-specific because reference standards, delivery mechanisms, and decisions differ from systemic RLT (6, 42, 52, 79, 86, 88–90).

⁹⁰Y studies provide secondary-platform evidence for quantitative planning, dose-toxicity linkage, and workflow automation; however, these findings should not be directly extrapolated to systemic RLT (6, 42, 52, 86, 88–90).

### 8.5 Longitudinal monitoring and cycle-by-cycle adaptation

Response-adaptive RPT requires a longitudinal loop in which early post-therapy imaging, absorbed dose, laboratory trends, symptoms, and tumor response update the risk-benefit balance prior to the next treatment cycle. Possible actions include continuing or delaying treatment, reducing or escalating the administered activity, altering the treatment interval, adding supportive care, or stopping the therapy. Current AI studies rarely test these actions prospectively and usually remain at the level of association, prediction, segmentation, or dose estimation (20–22, 44, 53–55, 101, 102).

The near-term opportunity is human-supervised decision support rather than a fully autonomous adaptation. A useful system should display inputs, uncertainty, reference standards, validation settings, and proposed clinical actions, and flag cases outside the development domain, such as different tracers, scanners, intervals, disease distributions, or toxicity phenotypes (17–19).

Between cycles, target expression, delivered activity and dose, organ function, marrow reserve, response, symptoms, and uncertainty form a changing, only partially observed patient state. A constrained adaptive policy could recommend continuation, delay, activity, or interval modification, supportive care, switching, or cessation while preserving clinician override. Its objective would have to balance competing utilities for efficacy, toxicity, quality of life, and resource burden under explicit safety constraints. Dynamic treatment regimes, offline or constrained reinforcement learning, model-predictive control, and off-policy evaluation are investigational: no mapped clinical RPT report prospectively validated such a policy, and historical treatment choices are confounded by indication. Simulation and silent prospective evaluation should precede an interventional treatment-changing study.

### 8.6 Theranostic digital twins: conceptual value and evidentiary limits

Theranostic digital twin papers frame patients as longitudinal systems integrating imaging, pharmacokinetics, dosimetry, laboratory data, toxicity, response, and radiobiology to simulate alternative strategies. In the mapped literature, they are conceptual or component-level roadmaps, not external, prospective, or decision-impact validation. Clinical evaluation would require validated modules, propagated uncertainty, prospective updating, auditability, and comparison of recommended actions with usual care (20–22, 44).

### 8.7 Radiobiology and mechanistic pharmacokinetics

The absorbed dose does not fully represent the biological effect. Relevant inputs include the dose rate, repair and repopulation, fractionation across cycles, linear-energy transfer, relative biological effectiveness (RBE), biologically effective dose, equivalent uniform dose, heterogeneity, radiosensitivity, tumor-control probability, and normal-tissue-complication probability. Alpha emitters additionally require microdosimetry, subcellular localization, daughter redistribution, and platform-specific RBE. Mechanistic, physiologically based pharmacokinetic (PBPK), and population-pharmacokinetic models can complement learned prediction and support sparse sampling or virtual patients, but their parameters, calibration, and clinical action thresholds require prospective validation (99, 103, 104).

## 9 Clinical translation and implementation

Translation should proceed according to the intended use: supervised segmentation or reporting, quantitative substitution within a specified margin, and treatment-changing policy evaluation. Each level requires task-appropriate reference standards, genuinely held-out testing, uncertainty and failure handling, workflow comparison, and progressively stronger clinical endpoints. The sections below consolidate the cross-cutting requirements rather than repeating them within each platform (8, 11, 16–19, 32, 33, 36).

### 9.1 Validation requirements before treatment-changing use

For treatment-changing use, validation must match the intended decision. A model used to prioritize contour review should demonstrate that it detects relevant failures and reduces the workflow time without degrading activity quantification. A model used for absorbed-dose estimation should be compared with an appropriate reference, such as serial quantitative imaging with MIRD calculations, voxel S values, dose kernel convolution or Monte Carlo simulation. A model used for activity escalation, de-escalation, delay, or cessation requires prospective evidence that the recommendation improves a prespecified clinical, safety, patient burden, or operational outcome without compromising efficacy (8, 11, 17–19, 32, 33, 36).

Internal cross-validation, random splits, and pooled multicenter developments do not establish generalizability. RPT performance may shift with the scanner, reconstruction, institution, population, imaging time, segmentation convention, and dosimetry method. External validation should be reserved for genuinely held-out centers, datasets, or appropriate temporal cohorts (17–19, 32, 33, 36).

### 9.2 Quantitative standards, uncertainty, and QA

Quantitative QA includes scanner calibration, corrections, activity recovery, registration, segmentation, kinetic fitting, and dose conversion. It should include biological plausibility checks, outlier flags, prior-cycle comparisons, and reconciliation of discordant estimates. Uncertainty must remain measurable: distinguish aleatoric from epistemic components; report calibration intercept and slope, interval coverage, repeatability, localized segmentation error, and out-of-distribution performance; and propagate relevant components to dose and action thresholds. Conformal methods may provide marginal coverage under exchangeability but not subgroup conditional guarantees under shifts. Selective prediction should report coverage–risk curves, deferral frequency, performance among non-deferred cases, false alert burden, and the safety of the reference workflow fallback (8–11, 17–19, 105).

### 9.3 Workflow integration and interoperability

Workflow-ready modules should preserve DICOM objects, calibration metadata, and audit trails across PACS, dosimetry, planning, laboratory, and record systems. Evaluation should report corrections, failures, abstention, time, and downstream testing, with genuinely site-held-out assessment, scanner and reconstruction harmonization, site-stratified or hierarchical analyses when appropriate, and prespecified local recalibration. Federated learning protects data locality but does not remove scanner, protocol, label, population, or missing-data shift. Self-supervised pretraining, multimodal PET/CT or PET/MRI, longitudinal sequence models, missing-modality-robust fusion, foundation models, vision-language systems, and transfer across tracers remain enabling methods until tested on a specified RPT task, reference standard, external cohort, and clinical action; PET/CT representation in current vision-language corpora is limited (106). Synthetic data require quantitative-fidelity, contamination, and memorization checks plus independent real-patient testing. Performance should be examined by center and, when sample size and data quality permit, by sex, age, ancestry, or race and ethnicity, geography, disease burden, scanner class, and resource setting (13, 16, 21, 24, 107).

### 9.4 Documentation and lifecycle requirements

Documentation should specify the intended use, provenance, inputs and outputs, calibration, failure modes, uncertainty, human review, cybersecurity, versioning, monitoring ownership, drift and recalibration triggers, override and incident logs, rollback, update, periodic revalidation, and decommissioning criteria. Workflow evaluation should measure hands-on and elapsed times, correction burden, failures, abstention, repeat processing or imaging, downstream testing, and patient visits. Economic evaluation should state the perspective, time horizon, and comparator; measure implementation and maintenance costs, professional time, avoided failures, cost per successfully completed dosimetry workflow, and downstream treatment consequences; and report quality-adjusted life-years when appropriate, incremental cost-effectiveness or cost-utility, and sensitivity analysis (17–19, 108).

### 9.5 Trial designs for clinical decision impact

The largest evidence gap is the decision impact, not another internal validation AUC, Dice score, or dose map agreement metric. Useful designs include prospective validation of AI dosimetry against predefined references, multicenter validation of selection models, pragmatic trials comparing AI-supported adaptive planning with usual care, and stepped-wedge implementation studies of the workflow, safety, and adoption of AI. Toxicity-aware protocols should predefine thresholds for laboratory decline, organ dose, marrow dose, treatment delay, and administered activity modification (17–19).

Evaluation should distinguish three stages: silent deployment that measures performance and failure without influencing care; workflow-substitution or equivalence studies that test whether a supervised module can replace a specified task within a prespecified margin; and interventional treatment-policy trials that test whether model-informed actions improve patient, safety, or resource outcomes.

Endpoints should be clinically interpretable, including treatment completion, grade 3/4 toxicity, renal decline, xerostomia, at least 50% decline in prostate-specific antigen (PSA50), biochemical response, molecular imaging response, Response Evaluation Criteria in Solid Tumors/PET Response Criteria in Solid Tumors (RECIST/PERCIST) response, PFS, OS, quality of life, and resource utilization. In early phase studies, decision concordance, calibration, uncertainty, and workflow time may be acceptable endpoints, but patient outcome benefits should not be implied until demonstrated (17–19).

## 10 Discussion: controversies, limitations, and evidence gaps

Four decision problems recur. Individualized activity requires reproducible dosimetry and actionable dose-response or dose-toxicity evidence; reduced-timepoint methods require tracer-, organ-, time-, and decision-specific agreement margins; lesion- and voxel-level maps add uncertainty that must provide value beyond organ means; and developmental metrics such as AUC, Dice, correlation, dose-map error, or workflow time do not establish improved treatment completion, toxicity, survival, quality of life, or resource use. These are reference-method equivalence or decision-impact questions, not algorithm competitions (1–11, 20, 32–38, 53–55, 86–92, 94–96, 101, 102, 109, 110).

### 10.1 Dataset limitations, leakage, generalizability, and cohort overlap

Generalizability depends on the tracer, platform, reconstruction, segmentation, imaging timing and reference dosimetry. Patient-level splitting is essential when multiple lesions, scans, or cycles arise from one patient. Lesion-level random splits can leak correlated observations across training and testing. Reused or overlapping institutional cohorts can also make apparently independent studies less independent than their citations suggest. A PSMA model may not transfer to SSTR PET, ^131^I imaging, ⁹⁰Y imaging, or alpha-emitter dosimetry without explicit testing (16–18, 32, 33).

### 10.2 Scope and limitations

This review uses a structured narrative design with descriptive report-level mapping. It was not designed for exhaustive systematic identification or meta-analysis, and no complete prospective screening or full-text exclusion log was available; those counts were not reconstructed. Primary selection was performed by M.B., while S.E.'s secondary verification was restricted to retained reports; excluded records were not independently reassessed, and eligible reports may have been missed. Heterogeneity in tasks, endpoints, references, and reporting precluded pooled estimates and a single risk-of-bias instrument. We therefore used task-appropriate descriptive characterization rather than a formal comparative risk-of-bias assessment. The six-report pilot was purposively selected to span task families and was intended to clarify the application of the domains; it was not a representative sample, reliability study, or validation of the synthesis. Six of 73 journal reports were available only through an official abstract or publisher preview, limiting extraction. Report counts may include related publications from overlapping cohorts. Full reports, technical or synthetic evaluations, direct conference abstracts, and contextual evidence were kept separate. Early-online availability near the July 18, 2026 endpoint may alter the distribution, and periodic updating will be necessary as new full reports supersede preliminary or abstract-only evidence. Conclusions apply to the mapped narrative set and are not prevalence estimates for the entire literature.

## 11 Research roadmap

A practical research roadmap should focus on converting technical feasibility into clinical decision support: better reference standards, standardized datasets, prospective validation, clinically meaningful endpoints, uncertainty-aware workflow integration, and evidence that AI-supported outputs improve management decisions (16–19).

### 11.1 Build standardized, multicenter theranostic datasets

The highest-yield infrastructure goal is a curated multicenter dataset linking diagnostic imaging, post-therapy quantitative imaging, laboratory values, administered activity, absorbed dose estimates, response and toxicity endpoints, and longitudinal outcomes. Shared acquisition, reconstruction, calibration, segmentation, and dosimetry metadata would allow models to be compared under common rules rather than isolated, single-center reports (16, 32, 33).

### 11.2 Establish dose-response and dose-toxicity consortia

AI-guided personalization will remain limited until absorbed dose estimates are linked to validated responses and toxicity relationships. Multicenter dose-response consortia should pool patient-, organ-, lesion-, and voxel-level dosimetry with standardized marrow, kidney, liver, lung, tumor, and other clinically relevant outcomes (53–55, 101).

### 11.3 Test adaptive treatment strategies prospectively

Future trials should test prespecified adaptive strategies, including response-adapted continuation or discontinuation, toxicity-aware delay, and activity escalation or de-escalation, only when target expression, organ reserve, uncertainty bounds, and protocol-defined safety constraints support these actions. Decision impact and clinician override should be documented prospectively (17–19, 53–55, 101).

### 11.4 Develop workflow-ready modular decision support

Rather than waiting for complete digital twins, investigators should build validated modules for immediate clinical problems, such as organ segmentation with automated QA, tumor burden dashboards, single-timepoint dosimetry calculators with uncertainty, marrow-risk flags, lesion tracking, and structured reports that distinguish measured from predicted values. Each module requires independent validation and clear communication of its limitations (30, 34, 36, 37, 53, 54, 110).

### 11.5 Keep emerging platforms proportionate but visible

CXCR4-targeted therapy, ^223^Ra, ^22^⁵Ac, and other alpha-emitting therapies, and Auger-electron therapies may eventually require AI-supported imaging, microdosimetry, and response adaptation. Current AI-specific evidence remains sparse or preliminary; therefore, these platforms should illustrate research needs rather than carry equal evidentiary weight to PSMA and SSTR/PRRT (45–48, 111).

### 11.6 Measure patient-centered and operational outcomes

Implementation should also measure patient-centered and operational outcomes, including fewer visits and scans, lower cumulative burden, preserved quality of life, fewer severe toxicities, faster reporting, better treatment completion, and more consistent, multidisciplinary decisions. Workflow metrics should be reported with the same rigor as model metrics (17–19).

## 12 Conclusion

Within the mapped narrative set, the evidence supports human-reviewed AI for segmentation, quantitative measurement, and selected dosimetry workflow tasks, but does not establish that AI-guided treatment selection or activity adaptation improves patient outcomes (30, 31, 34, 36, 37, 75, 80, 85, 90, 97, 110). Evidence characterization is narrative and descriptive rather than a formal comparative risk of bias assessment or pooled synthesis.

Priorities differ by platform: PSMA studies require external treatment-effect and toxicity models linked to prespecified actions; SSTR/PRRT requires reference-dosimetry equivalence and prospective dose-response or dose-toxicity cohorts; radioiodine requires broader patient-separated external and prospective validation, calibration, and decision-impact testing, with administered activity or post-therapy imaging excluded from models claimed for pretreatment selection; ⁹⁰Y radioembolization requires harmonized quantitative imaging and multicenter response/toxicity validation; and FAP- and alpha-targeted therapies first require platform-specific quantitative and dosimetric validation before AI-guided treatment policies are tested (27, 28, 39–43, 45–48, 50, 51, 59–79, 84, 88–90, 96, 99, 103, 104).

Treatment-changing use will require standardized quantitative imaging, appropriate dosimetry comparators, multicenter and prospective validation, calibrated uncertainty, clinically established dose-response or dose-toxicity relationships, and a pre-specified evaluation of decision impact (8, 11, 17–19, 33, 36, 53, 55).
